# Impact Damage of Aluminum-Composite Sandwich Panels and Constituents

**DOI:** 10.3390/ma18092105

**Published:** 2025-05-03

**Authors:** Shun-Fa Hwang, Ming-Yi Wu

**Affiliations:** Department of Mechanical Engineering, National Yunlin University of Science and Technology, 123 University Road, Sec. 3, Yunlin 64002, Taiwan; m11011032@yuntech.edu.tw

**Keywords:** metal-composite sandwich panel, low-velocity impact, finite element analysis, aluminum alloy, composite material

## Abstract

This study investigates the impact failure behavior of aluminum-composite sandwich panels, aluminum sheets, and composite laminates. Aluminum alloy sheets possess excellent ductility and plasticity, while carbon fiber composite sheets exhibit high strength, high rigidity, and superior heat resistance. The sandwich panel structure, composed of two layers of aluminum alloy sheets and a central carbon fiber composite sheet, offers the advantages of being lightweight and high strength. These three types of specimens were subjected to impact energies of 15 J, 25 J, and 50 J. The numerical simulations employ LS-DYNA finite element software, with additional investigations into the energy absorption characteristics, which were employed and compared with the experiment. The experimental results indicate that aluminum alloy sheets only exhibit indentation under all three impact energies. Carbon fiber composite sheets sustain damage without penetration at 15 J but experience penetration failure at 25 J and 50 J. Aluminum-composite sandwich panels exhibit greater resistance to failure as compared to carbon fiber composites. At 15 J and 25 J, the top aluminum layer shows indentation, while the bottom aluminum layer develops cracks. At 50 J, complete penetration occurs. A comparison of damage morphology and force–time curves shows good agreement between the experimental and simulation results. While the carbon fiber composite plate exhibits the highest SEA, it also has the largest damage diameter, indicating more severe damage. In contrast, the aluminum alloy panel has the lowest specific energy absorption (SEA) due to its high weight. The aluminum-composite sandwich panel demonstrates intermediate performance in both damage diameter and SEA, striking a balance between the other two specimens.

## 1. Introduction

With advancements in technology, industries such as aerospace, automotive, and marine engineering have increasingly pursued lightweight materials to enhance performance and efficiency. Carbon fiber-reinforced composites have emerged as a widely used solution due to their low density and high rigidity. These materials exhibit exceptional mechanical properties, being composed of carbon fibers embedded within a polymer matrix to form a strong composite structure. However, most composite materials are inherently brittle and susceptible to impact damage. Unlike metals, which absorb energy through plastic deformation, composites dissipate impact energy through internal damage mechanisms, such as fiber breakage, matrix cracking, and delamination. To improve the impact resistance of composite materials, hybrid structures integrating metals and composites have been developed. One such solution is the metal-composite sandwich panel or fiber metal laminate (FML), a three-layer structure consisting of metal sheets enclosing a composite core. This design leverages the ductility and corrosion resistance of metals alongside the strength and stiffness of composites, creating a structurally efficient and impact-resistant material. Aluminum, titanium, and magnesium are three common types of metals for FML, and the most popular one is aluminum. Aluminum has been combined with different composite materials into some famous FMLs, for example, GLARE (glass fibers), CARALL (carbon fibers), and ARALL (aramid fibers). Among them, GLARE has been studied and applied in airframe construction, fuselage frames, and cargo bay floors [1]. On the other hand, CARALL (carbon fibers and aluminum) is seldom studied.

For AA5083-H116 aluminum alloy plates, an analytical perforation model and a nonlinear finite element simulation were established to predict their perforation behavior under ballistic limit velocities [2]. The same alloy plates were experimentally and numerically investigated by Grytten et al. [3] on the quasi-static perforation with varying plate thickness, boundary conditions, punch diameters, and nose shapes. The static impact behavior of aluminum helideck structures to access offshore installations was an experimental examination [4]. Morin et al. [5] discussed the behavior and failure of stiffened plates made of aluminum alloy AA6082-T6 by both the quasi-static and low-velocity impact loadings and found that the strain rate and inertia effects were negligible. For recent works, Liu et al. [6] presented a material failure criterion, which was the combination of the revised Bressan–Williams–Hill local instability criterion and damage evolution model, to predict their fracture behaviors under different nose shapes of the impactor. As compared to experimental results, the failure criterion had good agreement on the onset of fracture with even coarse meshes. Shi et al. [7] proposed analytical algebraic expressions for predicting the amount of permanent indentation of aluminum panels under impact by a spherical object. For polyurea-coated aluminum plates, Xia et al. [8] used hyperelastic and viscoelastic constitutive models for polyurea and a modified Johnson–Cook model for aluminum to simulate their impact responses. Their results indicated that the simulation results of impact peak force, energy absorption, and deformation were within 5% of the experimental results.

Similarly, for fiber-reinforced composite laminates, extensive research has been conducted on the low-velocity impact behavior [9,10,11,12,13,14,15,16]. For unidirectional fiber-reinforced composite laminates, the main topic was the effect of the stacking sequence on impact behavior [9,10,12], and the quasi-isotropic laminates could provide better resistance to impact. Caminero et al. [9] also discussed the thickness effect, and Zhou et al. [12] considered the residual tensile strength of CFRP laminates after impact. Ouyang et al. [11] focused on the effect of matrix cracks on delamination during impact and indicated that the delamination was easily created around the matrix cracks. For braided composite panels under impact, the effect of the stacking sequence was experimentally studied by Wu et al. [14], and the effect of thickness was investigated by Ge et al. [13]. The temperature effect on the impact behavior of braided composite laminates was considered by Khashaba et al. [15]. Krollmann et al. [16] investigated the impact and post-impact compression properties of hybrid-matrix laminates, in which carbon fiber-reinforced elastomer layers were combined with conventional carbon fiber-reinforced epoxy layers.

For aluminum-composite sandwich panels, Bienias and Jakubczak [17] proposed five damage states to describe the impact behavior, and they included internal degradation and plastic deformation, initiation of new cracks at a fiber/matrix interface and delamination, the initiation and growth of cracks in the metal layers, penetration of the structure and, finally, the withdrawal of the impactor. Drozdziel et al. [18] demonstrated that the damage mechanism of fiber metal laminates with thin-ply composites did not significantly differ from that with a conventional composite ply thickness and confirmed the use of thin-ply fiber metal laminates. Yu et al. [19] revealed that the carbon fiber aluminum laminates had better impact resistance than the glass fiber counterparts. Furthermore, the increase in the yield strength of aluminum alloy could improve the impact resistance of carbon fiber aluminum laminates. Jakubczak et al. [20] used both experimental tests and numerical simulations to find that the main damage modes of fiber metal laminates under low-velocity impact were matrix fracture, fiber cracking, and delamination. Li et al. [21] proposed a novel hybrid laminate by inserting elastomer layers into conventional fiber metal laminates, and these novel hybrid laminates could efficiently absorb more impact energy. Yao et al. [22] discussed the influence of impactor shape on the low-velocity impact behavior of fiber metal laminates by both experiment and simulation.

Aluminum-composite sandwich panels are a relatively new type of fiber metal laminates, which combine the good characteristics of metals and composite materials. This sandwich panel possesses lightweight, better fatigue behavior, and corrosion resistance from composite materials and obtains higher ductility and bearing strength from metals. They are a good substitute for traditional metals used in aircraft and the aerospace industry. Therefore, it is necessary to evaluate their damage condition from collision during the process of maintenance and operation. Especially, the advantage of aluminum-composite sandwich panels needs to be identified as compared to the two original materials. This study addresses this gap by systematically investigating the failure modes, force–time response, and energy absorption characteristics of these three material configurations under varying impact energies. Furthermore, the numerical simulation is also proposed to compare with the experiment and verify its application for low-velocity impact.

## 2. Thermoplastic Composite Material and Thermoforming Process

**Figure 1:**
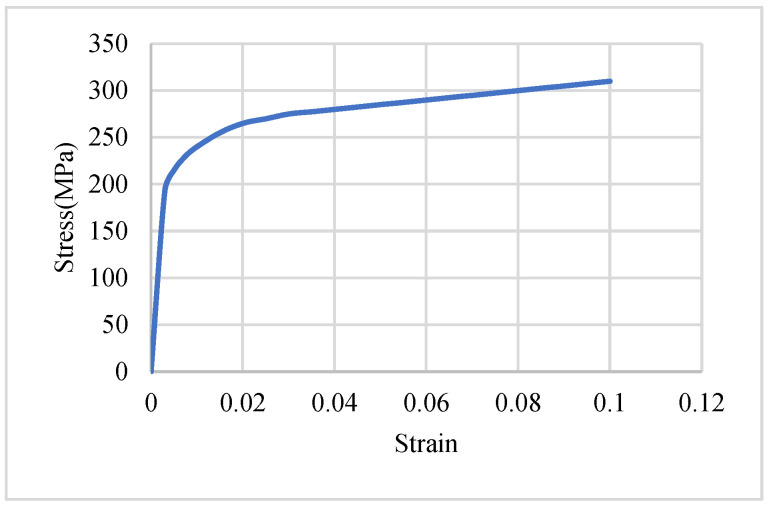
The stress–strain curve of 5052-H32 aluminum alloy [23].

The materials utilized in this study include 5052-H32 aluminum alloy sheets, unidirectional carbon fiber composites, and aluminum-composite sandwich panels. The 5052-H32 aluminum alloy, provided by Fabow Industrial Co., Ltd (Taiwan), is a strain-hardened alloy with moderate strength, excellent corrosion resistance, and high fatigue strength. The material’s stress–strain curve and mechanical properties are presented in Figure 1 and Table 1, respectively.

The unidirectional carbon fiber prepreg used in this study was manufactured by Formosa Plastics Corporation, with the fiber type designated as TC-36P (12 K). The fiber areal weight (FAW) is 150 g/m^2^, and the measured fiber weight fraction is 62%. The detailed material properties of the fabricated composite are provided in Table 2.

Three types of specimens were prepared: aluminum alloy sheets, carbon fiber composite laminates, and aluminum-composite sandwich panels, each with dimensions of 110 × 110 mm. Due to manufacturing constraints, the exact thicknesses varied slightly, with aluminum sheets at 2 mm, carbon fiber composites at 1.92 mm, and sandwich panels at 1.84 mm.

The aluminum alloy specimens were pre-cut by Fabow Industrial Co., Ltd., while the composite laminates and sandwich panels were fabricated using a hot-press technique. Carbon fiber composite laminates were prepared by stacking twelve layers of unidirectional prepreg sheets in a [(0°/90°)_3_]_s_ configuration. During stacking, a cylindrical roller was used to remove trapped air and prevent void formation. The laminates were then enclosed in release films and placed between aluminum plates coated with a mold release agent before undergoing hot pressing at 140 °C for 30 min without pressure, followed by an additional 1 h curing stage under 50 kgf pressure. The specimens were cooled to room temperature over three hours before final trimming with a diamond saw.

Aluminum-composite sandwich panels were fabricated similarly, with aluminum alloy face sheets (0.6 mm thick) bonded to a four-layer carbon fiber core ([0°/90°]_s_, 0.64 mm thick). Before bonding, the aluminum sheets were sanded to enhance adhesion. 

Impact tests were conducted using an impact testing machine equipped with a spectrum analyzer. The force exerted during impact was calculated using an accelerometer mounted on the impactor, while the damage diameter was measured using a vernier caliper. During the impact process, the accelerometer generated acceleration signals, which were transmitted to the spectrum analyzer. The acceleration–time curve was derived from these signals, and the force–time curve was obtained by multiplying the acceleration by the impactor mass. The impact energy levels tested were 15 J, 25 J, and 50 J, and the impactor had a mass of 7.918 kg and a diameter of 16 mm. By adjusting the drop height, one can obtain the impact velocities of 1.946, 2.513, and 3.554 m/s for the three impact energies, respectively. The reason for choosing these three impact energies was to have different damage phenomena for the three types of specimens.

## 3. Finite Element Simulation

**Figure 2:**
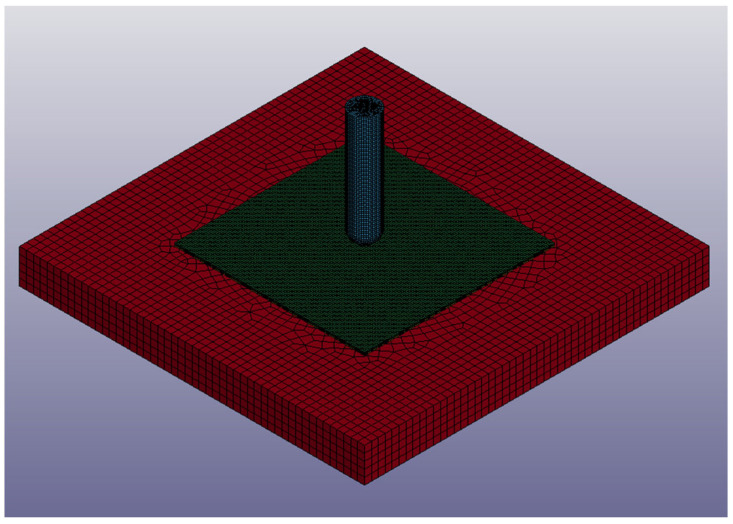
Mesh of the impact model.

In this study, LS-DYNA R15 software was employed to simulate low-velocity impact behavior. The simulation model included the impactor, the fixture, and the specimen. The mesh utilized two element types: eight-node solid elements for the impactor, fixture, and aluminum sheets, and four-node shell elements for the composite part. The element sizes were 1 × 1 × 1 mm for the solid element and 1 × 1 mm for the shell element. The mesh is shown in Figure 2. In the carbon fiber composite specimen, two shell elements represented the composite laminate, with six integration points per shell, totaling twelve points. Each integration point had a thickness of 0.16 mm, matching the experimental [(0°/90°)_3_]_s_ stacking sequence. In the aluminum-composite sandwich panel model, aluminum sheets were represented by solid elements, while the composite core was modeled using a single shell element with four integration points, each 0.16 mm thick, reflecting the [0°/90°]_s_ stacking configuration. 

To match the experiment and reduce the simulation time, the impactor with the corresponding velocities for different impact energies was released just above the specimen. In the impact experiment, rubber clamps were used at the four corners of the specimen to constrain its movement. In the simulation, this effect was replicated by constraining the corner nodes. Furthermore, contact interactions were enforced between different parts of the model to prevent penetration.

The impactor and fixture were modeled as rigid bodies, as their deformation was not considered. The impactor was set to move vertically while restricting rotational motion, whereas the fixture was completely fixed. The material properties used for these two parts had a density of 6.045 g/cm^3^, a Young’s modulus of 210 GPa, and a Poisson’s ratio of 0.3. Since impact energy is influenced by the impactor mass, the density of the impactor was adjusted based on its volume to ensure that its total mass remained at 7.918 kg, consistent with the experimental setup.

The aluminum alloy sheets were modeled using the *MAT_24_PIECEWISE_LINEAR_PLASTICITY material card, which defines elastoplastic behavior. The material properties assigned to this model are shown in Table 1 and Figure 1. In addition to these properties, the failure strain for the aluminum alloy sheets was set to 0.6 in the simulation. When the maximum plastic strain reached 0.6, the element was considered to have failed and was removed from the calculation. For the composite sheets, the *MAT_54_ENHANCED_COMPOSITE_DAMAGE material card was used. This model was specifically designed for unidirectional fiber-reinforced composites. The material card requires definitions for material properties, failure criteria, element orientation, and failure strain, with the material properties detailed in Table 2. The failure criterion employed in *MAT_54_ENHANCED_COMPOSITE_DAMAGE follows the Chang–Chang failure model [25]. This failure criterion is categorized into fiber direction tensile failure, fiber direction compressive failure, transverse direction matrix tensile-shear failure, and transverse direction matrix compressive-shear failure. In the simulation, if any element exceeded all four failure criteria, it was considered failed and was no longer included in further calculations. If just one failure criterion was fulfilled, parts of the mechanical properties were set to zero, and the element was still active.

In the aluminum-composite sandwich panel simulation, the model consisted of an upper aluminum alloy sheet, a central composite sheet, and a lower aluminum alloy sheet. To simulate the bonding and failure behavior between these sheets, the tie-and-break condition was judged according to the interfacial stresses as the following equation.

(1) $${\left(\frac{\left|\sigma_{n}\right|}{NFLS}\right)}^{2}+{\left(\frac{\left|\sigma_{s}\right|}{SFLS}\right)}^{2}\geq 1$$

where $\sigma_{n}$ is the interfacial normal stress, $\sigma_{s}$ is the interfacial shear stress, and *NFLS* and *SFLS* are the corresponding interfacial strengths. When the computed value reached or exceeded 1, interfacial failure occurred. The normal and shear strengths were set to 8.8 MPa and 54 MPa, respectively.

## 4. Results and Discussion

In this study, the experimental study involved conducting impact tests on aluminum alloy sheets, carbon fiber composite sheets, and aluminum-composite sandwich panels under three impact energy levels: 15 J, 25 J, and 50 J. A total of nine types of experiments were conducted. To ensure consistency and repeatability, each type of experiment was performed three times. The damage modes observed in the experiments included indentation, damage without full penetration, and complete penetration, depending on the impact energy level.

**Figure 3:**
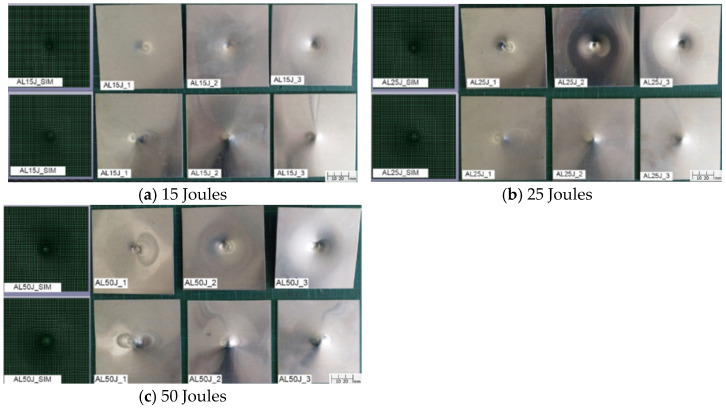
Simulation and experiment damage of aluminum specimens under 15 J, 25 J, and 50 J.

**Figure 4:**
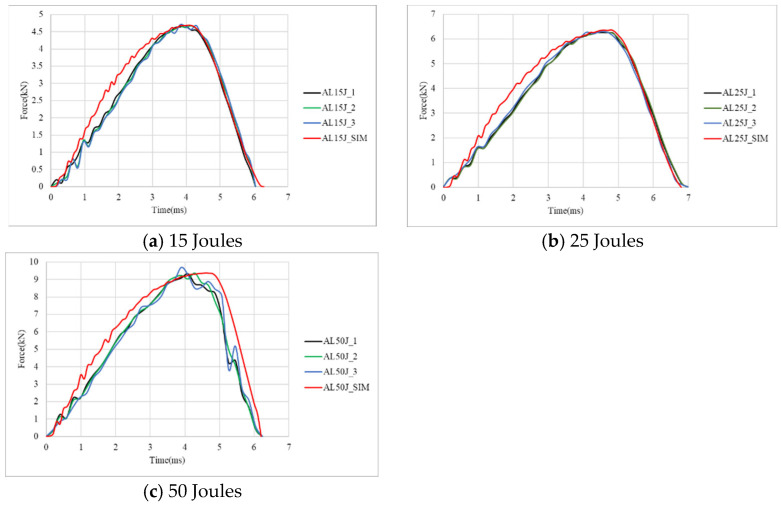
Simulation and experiment force–time curves of aluminum specimens under 15 J, 25 J, and 50 J.

Figure 3 illustrates the damage conditions of aluminum alloy sheets from both the simulation and repeated experiment under different impact energies, showing both top and bottom views with a full size of 110 × 110 mm. The damage results indicate that under impact energies of 15 J, 25 J, and 50 J, the aluminum alloy sheets only exhibit indentation without cracking or penetration. Due to their high ductility and high thickness, the aluminum alloy sheets were indented without failure, even under the impact of 50 J. The simulation also exhibits similar damage conditions, and the comparison of the predicted damage diameters with the average measured results is listed in Table 3, in which the standard deviation is also displayed in some average values. As shown, the maximum difference between the simulation and the experiment is only 3.6%. Furthermore, both results indicate that the damage diameter increases with the increase in impact energy. Figure 4 presents the corresponding force–time curves. The force–time curves for all three impact energy levels exhibit smooth and symmetrical profiles, indicating the absence of severe damage. As the impact energy increases, both the peak force and damage diameter increase accordingly, as listed in Table 3. The experimental results show that the average peak forces are 4.68 kN at 15 J, 6.26 kN at 25 J, and 9.46 kN at 50 J, with which the simulation has very good agreement. As listed in Table 3, the comparison of the impact time between the simulation and the experiment shows only minor discrepancies. It is interesting to note that at 25 J, the impact duration is longer. This is likely due to the indentation shape closely matching the impactor profile, leading to prolonged contact duration. All these results verify the impact simulation for the aluminum specimens.

**Figure 5:**
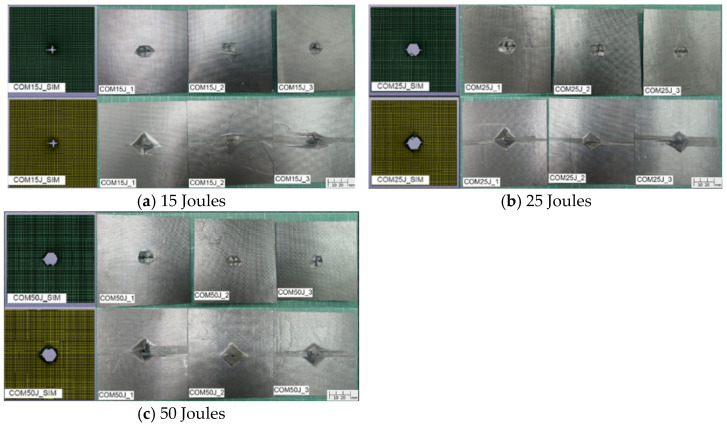
Simulation and experiment damage of carbon fiber composite specimens under 15 J, 25 J, and 50 J.

Figure 5 demonstrates the damage profile of carbon fiber composite specimens from both the simulation and repeated experiment under different impact energy levels, showing the top and bottom views with a full size of 110 × 110 mm. The results reveal that under a 15 J impact, the composite sheet sustains a fracture but does not experience full penetration. However, at 25 J and 50 J impact energies, penetration failure occurs. Compared to aluminum alloy sheets, carbon fiber composite sheets exhibit a higher susceptibility to damage. Although carbon fiber composites possess superior strength and stiffness, they also exhibit greater brittleness. When subjected to impact loading, this material is prone to damage, such as fiber breakage, matrix cracking, and delamination. Delamination between different layers is particularly evident on the backside of the impacted specimens, further exacerbating structural failure. The average damage diameters of the experiments under different impact energies are shown in Table 4 and compared with the simulation results. At 25 J and 50 J, the specimens were penetrated and had close damage diameters that were larger than those at 15 J. The diameters from the simulation only have 1.55% to 4.6% errors with the experiment values. A comparison of the failure morphology reveals slight differences between the experimental and simulation results. In the experimental specimens, significant delamination is observed on the backside of the impacted area. However, due to computational efficiency considerations, the simulation model utilizes only two shell elements to represent the composite sheet, preventing the accurate depiction of single-layer delamination.

**Figure 6:**
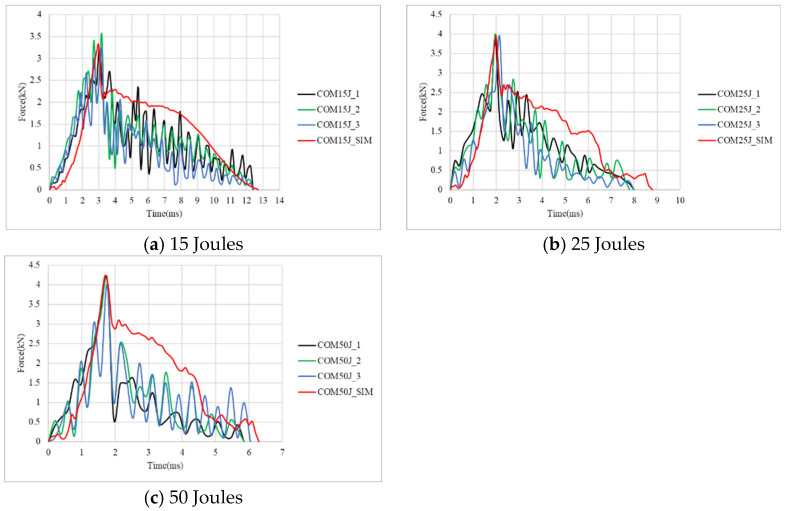
Simulation and experiment force–time curves of carbon fiber composite specimens under 15 J, 25 J, and 50 J.

Figure 6 presents the corresponding force–time curves from both the simulation and experiment. The force–time curves exhibit a common trend across all three impact energy levels: after reaching the peak force, a sharp decline is observed. This phenomenon indicates the initiation of severe internal damage, including fiber fracture, matrix failure, and interfacial delamination, which significantly reduces the specimen’s ability to withstand additional loading from the impactor. At 15 J, the composite specimen was not penetrated, such that the impact duration time is the longest. With the increase in impact energy, the impact time becomes shorter, as shown in Table 4. It is observed that beyond a certain impact energy threshold, the increase in peak force and damage diameter becomes less significant. This phenomenon occurs because, at 25 J, the composite sheet has already experienced full penetration, limiting its ability to further resist impact forces. Consequently, even when the impact energy is increased to 50 J, the peak force and damage diameter remain relatively unchanged. Additionally, as the impact energy increases, the impact duration decreases correspondingly. From Figure 6 and Table 4, the peak force from the simulation is less than 1.92% of the experiment, and the impact time has a maximum error of 9.2%. Therefore, the finite element simulation for the impact behavior of composite specimens has reliable results.

**Figure 7:**
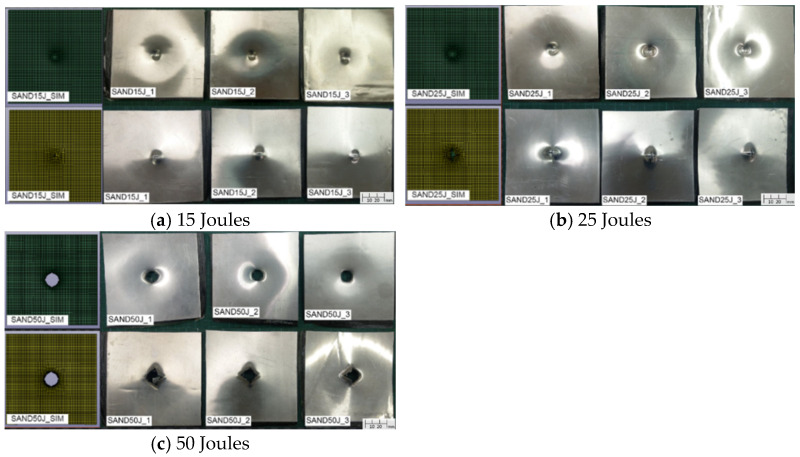
Simulation and experiment damage of sandwich specimens under 15 J, 25 J, and 50 J.

**Figure 8:**
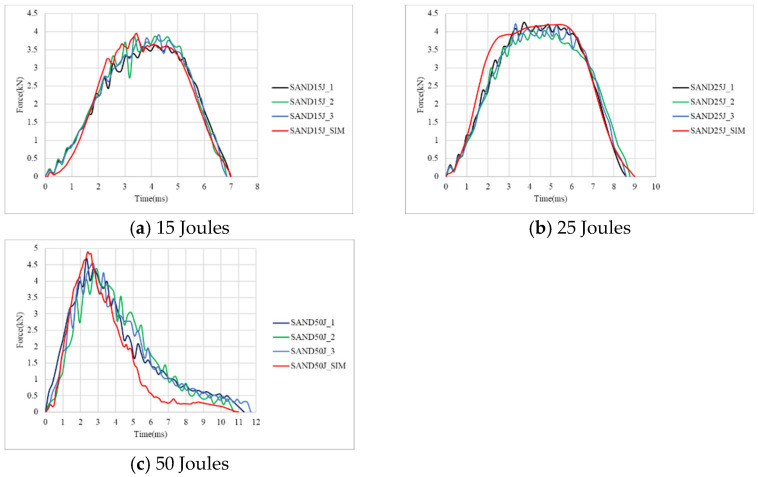
Simulation and experiment force–time curves of sandwich specimens under 15 J, 25 J, and 50 J.

As for the aluminum-composite sandwich specimens, the damage morphology under different impact energies, with the top and bottom views displayed separately with the full size of 110 × 110 mm, is shown in Figure 7. Figure 8 presents the corresponding force–time curves. The results indicate that under a 15 J impact, the top aluminum alloy layer exhibits indentation while the bottom aluminum layer develops minor cracks. At 25 J, the top aluminum layer continues to indent while the bottom aluminum layer develops more significant cracks. At 50 J, complete penetration occurs. Compared to carbon fiber composite specimens, aluminum-composite sandwich panels demonstrate greater resistance to penetration failure due to the plasticity of the aluminum alloy layers, which absorb impact energy through deformation. The force–time curves for 15 J and 25 J impacts exhibit smoother and more symmetrical profiles, reflecting the high plasticity of aluminum alloy and the absence of penetration failure. However, at 50 J, penetration failure occurs, resulting in a sharp force drop after reaching the peak load, as the specimen can no longer sustain the impact energy.

As listed in Table 5, as the impact energy increases, both peak force and impact duration time increase proportionally. For the damage diameter, there is a least value at 15 J, while the two diameters at 25 J and 50 J are very close. The close value on damage diameter may indicate the whole indentation of the impactor, even though there is no penetration at 25 J. Observing the failure deformation reveals a strong agreement between experimental and simulation results. Regarding the force–time curves, the discrepancies between the experimental and simulated curves are also minimal. The maximum error is just 8.6% for the peak force at 50 J.

**Figure 9:**
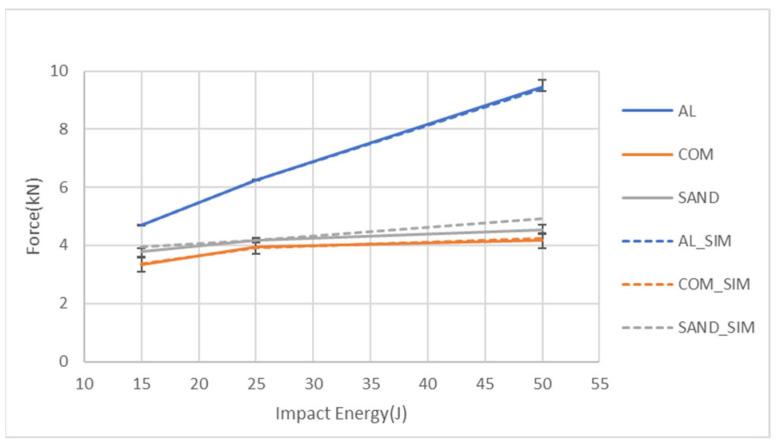
Comparison of the peak forces for the types of specimens under three impact energies from both the simulation and the experiment.

**Figure 10:**
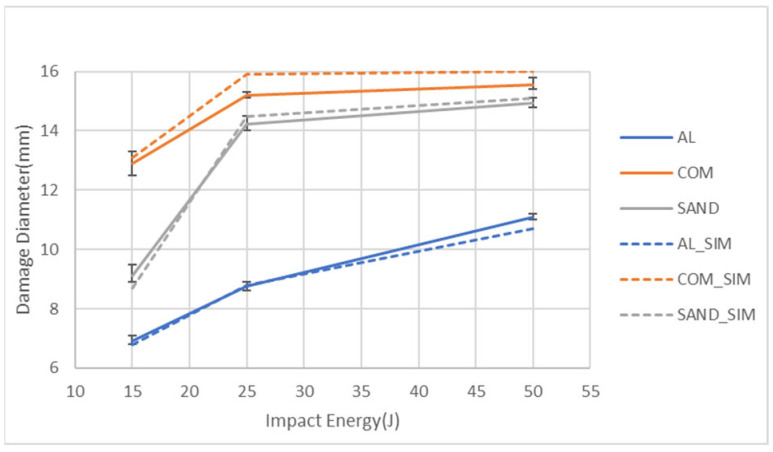
Comparison of the damage diameters for the types of specimens under three impact energies from both the simulation and the experiment.

Figure 9 presents a comparison of the peak force values obtained from the experiments and simulations for the three specimen types under different impact energy levels. The figure demonstrates a strong consistency between the simulation value and the experimental result, with error bars. Additionally, it can be observed that for all impact energies, the aluminum alloy sheet exhibits the highest peak force, and this force significantly increases with the impact energy. The peak force of the aluminum-composite sandwich panel is slightly higher than that of the carbon fiber composite sheet. In addition, the increase in impact energy does not increase the peak force of these two types of specimens much due to the damage of the composite part. Figure 10 compares the damage diameters obtained from the experiments and simulations for the three specimen types under different impact energy levels. The figure shows a strong correlation between the experimental and simulated values. Additionally, it can be observed that the carbon fiber composite sheet exhibits the largest damage diameter, followed by the aluminum-composite sandwich panel, with the aluminum alloy sheet having the smallest damage diameter. Furthermore, the damage diameter of the aluminum specimen keeps increasing with the impact energy. For both the sandwich specimens and the composite specimens, the damage diameter significantly increases from 15 J to 25 J, while the damage diameters at 25 J and 50 J have very close values that are also close to the impactor diameter. The reason should be from the easy damage of the composite part.

**Figure 11:**
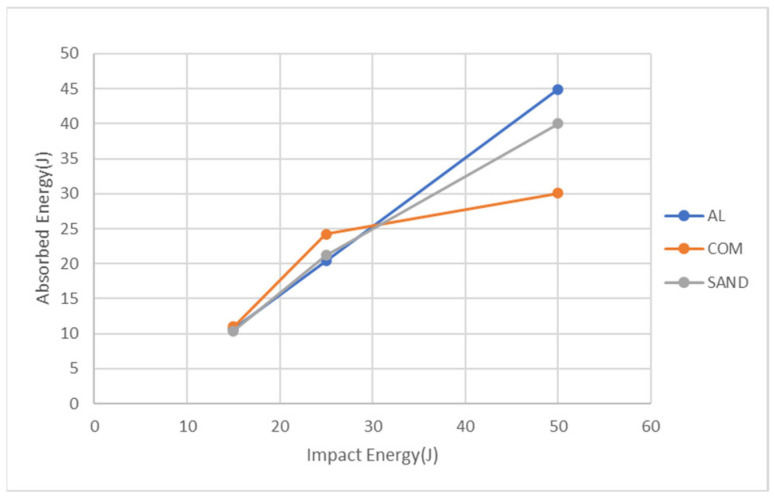
Predicted energy absorbed for the three types of specimens at the three impact energies.

Figure 11 presents the energy absorption from the simulation for the three specimen types at three impact energies. At 15 J, the carbon fiber composite sheet exhibits the highest energy absorption, reaching 11 J, followed by the aluminum alloy sheet at 10.7 J and the aluminum-composite sandwich panel at 10.4 J. At 25 J, the carbon fiber composite sheet exhibits the highest energy absorption at 24.2 J, followed by the aluminum-composite sandwich panel at 21.2 J and the aluminum alloy sheet at 20.4 J. At 50 J, the aluminum alloy sheet exhibits the highest energy absorption at 44.9 J, followed by the aluminum-composite sandwich panel at 40 J and the carbon fiber composite sheet at 30.1 J. From the above results, at impact energies of 15 J and 25 J, the easy damage of carbon fiber sheets absorbs the highest energy, while at 50 J, they absorb the least impact energy because of less further damage. For the other two types of specimens, the energy absorption maintains a linear increase with the impact energy, and at 50 J, the sandwich panel absorbs lower energy than the aluminum alloy sheet because the former is penetrated. All these suggest that while the carbon fiber composite sheet absorbs the most energy, its brittle nature makes it more prone to damage compared to the other materials, whereas the aluminum-composite sandwich panel leverages both the ductility of aluminum and the strength of the composite layer to balance impact resistance and energy absorption.

**Figure 12:**
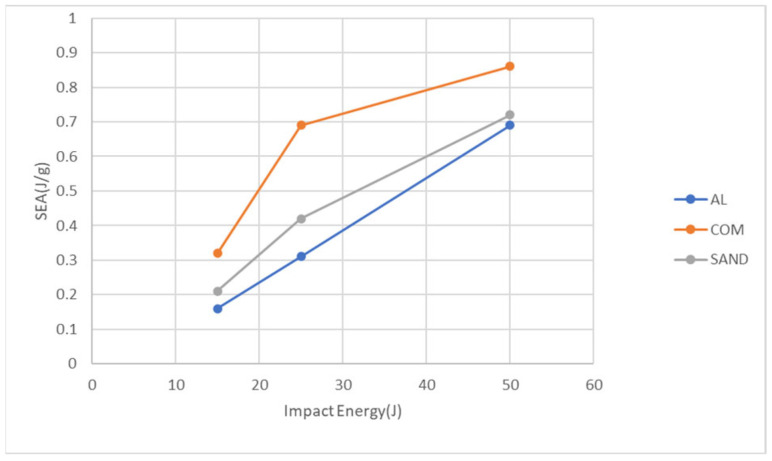
Predicted specific energy absorption for the three types of specimens at the three impact energies.

The total absorbed energy presented in Figure 11 was further normalized by dividing it by the specimen mass to determine the specific energy absorption (SEA), which represents the energy absorbed per unit mass. Figure 12 illustrates the specific energy absorption values for the three specimen types under different impact energy levels. The carbon fiber composite sheet exhibits the highest specific energy absorption, followed by the aluminum-composite sandwich panel, with the aluminum alloy sheet having the lowest value. Overall, while the carbon fiber composite sheet has the highest specific energy absorption, it also sustains the most severe damage and the lowest peak force, as shown in Figure 9 and Figure 10. The aluminum alloy sheet, due to its highest mass, exhibits the lowest specific energy absorption. However, due to its high ductility, it has the highest peak force and the lowest damage diameter. The aluminum-composite sandwich panel falls between the two, balancing both damage resistance and energy absorption efficiency. Although the sandwich panel’s damage diameter is larger than that of the aluminum alloy sheet, it also exhibits higher specific energy absorption. Conversely, while its specific energy absorption is lower than that of the carbon fiber composite sheet, its damage diameter is significantly smaller. In some applications, if one wants to absorb more impact energy with less mass, transfer less impact force, and maintain smaller damage or no penetration, the aluminum-composite sandwich (or FMLs) should be a good candidate.

## 5. Conclusions

This study conducted low-velocity impact experiments on three types of specimens: 5052-H32 aluminum alloy sheets, carbon fiber composite sheets made from unidirectional prepreg stacking, and aluminum-composite sandwich panels. The impact energies were set at 15 J, 25 J, and 50 J to investigate the failure morphology, damage diameter, and force–time response of each specimen under different impact conditions. Finite element analysis using LS-DYNA was performed to compare the experimental and simulation results, validating their consistency. The aluminum alloy sheets exhibited only indentation under all three impact energy levels (15 J, 25 J, and 50 J). Under an impact energy of 15 J, carbon fiber composite plates exhibit damage without complete penetration. However, at impact energies of 25 J and 50 J, the specimens undergo full penetration failure. Carbon fiber composite plates are more susceptible to damage. Under an impact energy of 15 J, the top aluminum alloy layer of the aluminum-composite sandwich panel exhibits indentation, while the bottom aluminum alloy layer develops minor cracks. At an impact energy of 25 J, the top layer remains indented, but the bottom layer experiences larger cracks. When subjected to 50 J impact energy, the specimen undergoes complete penetration failure.

A comparison of the three types of specimens reveals that, in terms of peak force, the aluminum alloy panel exhibits the highest peak force. Regarding the damage diameter, the carbon fiber composite plate exhibits the largest damage diameter. The aluminum-composite sandwich panel falls between the other two specimens in both peak force and damage diameter. By comparing the experimental results with LS-DYNA finite element analysis simulations, a strong consistency is observed in terms of damage diameter, failure morphology, peak force, and impact duration. This indicates that the simulation model demonstrates a high level of reliability and can effectively predict the outcomes observed in actual experiments. Furthermore, by analyzing the damage diameter and specific energy absorption (SEA), it is evident that while the carbon fiber composite plate exhibits the highest SEA, it also has the largest damage diameter. In contrast, the aluminum alloy panel has the lowest SEA. The aluminum-composite sandwich panel demonstrates intermediate performance in both damage diameter and SEA, striking a balance between the other two specimens.

## Figures and Tables

**Table 1:** Mechanical properties of 5052-H32 aluminum alloy [23].

Properties	Value	Unit
Density	2.68	g/cm^3^
Young’s modulus	70.3	GPa
Poisson’s ratio	0.33	-
Yield stress	193	MPa

**Table 2:** Mechanical properties of unidirectional composite material [24].

Property	Value	Unit
Density, *ρ*	1.5	g/cm^3^
Longitudinal Young’s modulus, *E*_11_	128	GPa
Transverse Young’s modulus, *E*_22_	8.5	GPa
Shear modulus, *G*_12_	5.69	GPa
Shear modulus, *G*_23_	3.44	GPa
Shear modulus, *G*_31_	5.69	GPa
Poisson’s ratio, *ν*_12_	0.2	-
Longitudinal tensile strength, *X_T_*	1340	MPa
Longitudinal compressive strength, *X_C_*	492	MPa
Transverse tensile strength, *Y_T_*	40	MPa
Transverse compressive strength, *Y_C_*	192	MPa
Shear strength, *S*	76	MPa

**Table 3:** Result comparison of aluminum specimens under 15 J, 25 J, and 50 J.

Specimen	Damage Diameter (mm)	Peak Force (kN)	Impact Time (ms)
AL15J_1	6.9	4.68	6.04
AL15J_2	6.8	4.67	6.04
AL15J_3	7.1	4.71	6.04
Average	6.93 ± 0.15	4.68 ± 0.02	6.04
Simulation	6.8 (−1.87%)	4.69 (0.21%)	6.29 (4.13%)
AL25J_1	8.8	6.26	7.02
AL25J_2	8.6	6.26	7.02
AL25J_3	8.9	6.27	7.02
Average	8.76 ± 0.15	6.26 ± 0.01	7.02
Simulation	8.8 (0.45%)	6.37 (1.75%)	6.8 (−3.13%)
AL50J_1	11.0	9.32	6.24
AL50J_2	11.1	9.36	6.24
AL50J_3	11.2	9.70	6.24
Average	11.1 ± 0.1	9.46 ± 0.21	6.24
Simulation	10.7 (−3.6%)	9.38 (−0.84%)	6.2 (0.64%)

**Table 4:** Result comparison of carbon fiber composite specimens under 15 J, 25 J, and 50 J.

Specimen	Damage Diameter (mm)	Peak Force (kN)	Impact Time (ms)
COM15J_1	13.3	3.21	12.4
COM15J_2	12.5	3.55	12.4
COM15J_3	12.9	3.23	12.4
Average	12.9 ± 0.4	3.33 ± 0.19	12.4
Simulation	13.1 (1.55%)	3.38 (1.5%)	12.68 (2.25%)
COM25J_1	15.1	3.90	8
COM25J_2	15.3	4.02	7.9
COM25J_3	15.2	3.95	8
Average	15.2 ± 0.1	3.95 ± 0.06	7.96
Simulation	15.9 (4.6%)	3.93 (−0.5%)	8.7 (9.2%)
COM50J_1	15.5	4.15	5.85
COM50J_2	15.4	4.10	5.85
COM50J_3	15.8	4.23	6.04
Average	15.56 ± 0.21	4.16 ± 0.07	5.91
Simulation	16 (2.82%)	4.24 (1.92%)	6.29 (6.4%)

**Table 5:** Result comparison of sandwich specimens under 15 J, 25 J, and 50 J.

Specimen	Damage Diameter (mm)	Peak Force (kN)	Impact Time (ms)
SAND15J_1	9	3.62	6.99
SAND15J_2	9.5	3.86	6.99
SAND15J_3	8.9	3.91	6.83
Average	9.13 ± 0.32	3.79 ± 0.16	6.88
Simulation	8.7 (−4.71%)	3.96 (4.48%)	7 (1.7%)
SAND25J_1	14.5	4.26	8.58
SAND25J_2	14	4.09	8.77
SAND25J_3	14.2	4.22	8.58
Average	14.23 ± 0.25	4.19 ± 0.09	8.64
Simulation	14.5 (1.89%)	4.19 (0%)	8.9 (3.0%)
SAND50J_1	14.8	4.7	11.31
SAND50J_2	15.1	4.37	10.72
SAND50J_3	14.9	4.54	11.7
Average	14.93 ± 0.15	4.53 ± 0.17	11.24
Simulation	15.1 (1.13%)	4.92 (8.6%)	11.02 (−1.9)

## Data Availability

Data are available on request from the corresponding author.

## References

[1] Muniyan V., Kumar V.V., Suyambulingam I., Priyadharshini S., Divakaran D., Rangappa S.M., Siengchin S. (2025). A review of recent advancements in the impact response of fiber metal laminates. Heliyon.

[2] Børvik T., Forrestal M.J., Hopperstad O.S., Warren T.L., Langseth M. (2009). Perforation of AA5083-H116 aluminium plates with conical-nose steel projectiles—Calculations. Int. J. Impact Eng..

[3] Grytten F., Børvik T., Hopperstad O.S., Langseth M. (2009). Quasi-static perforation of thin aluminium plates. Int. J. Impact Eng..

[4] Seo J.K., Park D.K., Jo S.W., Kim B.J., Park J.S. (2018). Experimental assessment of the structural behaviour of aluminium helideck structures under static impact loads. Ships Offshore Struct..

[5] Morin D., Kaarstad B.L., Skajaa B., Hopperstad O.S., Langseth M. (2017). Testing and modelling of stiffened aluminium panels subjected to quasi-static and low-velocity impact loading. Int. J. Impact Eng..

[6] Liu B., Chen C.T., Garbatov Y. (2022). Material failure criterion in the finite element analysis of aluminium alloy plates under low-velocity impact. Ocean Eng..

[7] Shi S., Lam N., Cui Y.W., Zhang L.H., Lu G.X., Gad E. (2022). Indentation into an aluminium panel by the impact of a rigid spherical object. Thin-Walled Struct..

[8] Xia Y., Shi Z.T., Zhou Q., Ao W.H. (2023). Numerical investigation on polyurea coated aluminum plate subjected to low velocity impact. Int. J. Impact Eng..

[9] Caminero M.A., García-Moreno I., Rodríguez G.P. (2017). Damage resistance of carbon fibre reinforced epoxy laminates subjected to low velocity impact: Effects of laminate thickness and ply-stacking sequence. Polym. Test..

[10] Long S.C., Chen C., Wang H., Yao X.H., Zhang X.Q. (2022). Distribution and propagation of matrix cracks within composite laminates under impact. Compos. Struct..

[11] Ouyang T., Sun W., Bao R., Tan R.M. (2021). Effects of matrix cracks on delamination of composite laminates subjected to low-velocity impact. Compos. Struct..

[12] Zhou J.W., Liao B., Shi Y., Zuo Y.G., Tuo H.I., Jia L.Y. (2019). Low-velocity impact behavior and residual tensile strength of CFRP laminates. Compos. Part B Eng..

[13] Ge X., Zhang P., Zhao F., Liu M., Liu J., Cheng Y. (2022). Experimental and numerical investigations on the dynamic response of woven carbon fiber reinforced thick composite laminates under low-velocity impact. Compos. Struct..

[14] Wu Z.Y., Wu C.J., Liu Y.S., Cheng X.Y., Hu X.D. (2020). Experimental study on the low-velocity impact response of braided composite panel: Effect of stacking sequence. Compos. Struct..

[15] Khashaba U.A., Othman R. (2017). Low-velocity impact of woven CFRE composites under different temperature levels. Int. J. Impact Eng..

[16] Krollmann J., Schreyer T., Veidt M., Drechsler K. (2019). Impact and post-impact properties of hybrid-matrix laminates based on carbon fiber-reinforced epoxy and elastomer subjected to low-velocity impacts. Compos. Struct..

[17] Bieniaś J., Jakubczak P. (2017). Impact damage growth in carbon fibre aluminium laminates. Compos. Struct..

[18] Droździel M., Jakubczak P., Bieniaś J. (2021). Low-velocity impact resistance of thin-ply in comparison with conventional aluminium-carbon laminates. Compos. Struct..

[19] Yu G.C., Wu L.Z., Ma L., Xiong J. (2015). Low velocity impact of carbon fiber aluminum laminates. Compos. Struct..

[20] Jakubczak P., Bieniaś J., Dadej K. (2020). Experimental and numerical investigation into the impact resistance of aluminium carbon laminates. Compos. Struct..

[21] Li Z.Y., Zhang J.Y., Jackstadt A., Kärger L. (2022). Low-velocity impact behavior of hybrid CFRP-elastomer-metal laminates in comparison with conventional fiber-metal laminates. Compos. Struct..

[22] Yao L., Wang C.Z., He W.T., Lu S.J., Xie D. (2019). Influence of impactor shape on low-velocity impact behavior of fiber metal laminates combined numerical and experimental approaches. Thin-Walled Struct..

[23] ASM Aerospace Specification Metals Inc. (2025). ASM Material Data Sheet.

[24] Wu C.Y. (2019). Study of Impact Energy Absorption on the Aluminum-Composite Hybrid Tubes. Master’s Thesis.

[25] Chang F.K., Chang K.Y. (1987). A progressive damage model for laminated composites containing stress concentration. J. Compos. Mater..

